# 3D Models of MBP, a Biologically Active Metabolite of Bisphenol A, in Human Estrogen Receptor α and Estrogen Receptor β

**DOI:** 10.1371/journal.pone.0046078

**Published:** 2012-10-04

**Authors:** Michael E. Baker, Charlie Chandsawangbhuwana

**Affiliations:** 1 Department of Medicine, University of California San Diego, La Jolla, California, United States of America; 2 Department of Bioengineering, University of California San Diego, La Jolla, California, United States of America; Ecole Normale Supérieure de Lyon, France

## Abstract

Bisphenol A [BPA] is a widely dispersed environmental chemical that is of much concern because the BPA monomer is a weak transcriptional activator of human estrogen receptor α [ERα] and ERβ in cell culture. A BPA metabolite, 4-methyl-2,4-bis(4-hydroxyphenyl)pent-1-ene [MBP], has transcriptional activity at nM concentrations, which is 1000-fold lower than the concentration for estrogenic activity of BPA, suggesting that MBP may be an environmental estrogen. To investigate the structural basis for the activity of MBP at nM concentrations and the lower activity of BPA for human ERα and ERβ, we constructed 3D models of human ERα and ERβ with MBP and BPA for comparison with estradiol in these ERs. These 3D models suggest that MBP, but not BPA, has key contacts with amino acids in human ERα and ERβ that are important in binding of estradiol by these receptors. Metabolism of BPA to MBP increases the spacing between two phenolic rings, resulting in contacts between MBP and ERα and ERβ that mimic those of estradiol with these ERs. Mutagenesis of residues on these ERs that contact the phenolic hydroxyls will provide a test for our 3D models. Other environmental chemicals containing two appropriately spaced phenolic rings and an aliphatic spacer instead of an estrogenic B and C ring also may bind to ERα or ERβ and interfere with normal estrogen physiology. This analysis also may be useful in designing novel chemicals for regulating the actions of human ERα and ERβ.

## Introduction

One consequence of our industrial society is the presence of novel environmental chemicals that disrupt normal physiological responses in humans, other vertebrates, as well as invertebrates [1], [2]. Many of these chemicals are small hydrophobic molecules that resemble steroids, thyroid hormone, retinoids and other lipophilic hormones and, as a result bind to their receptors in vertebrates [3], [4], [5], [6], [7]. Some of these chemicals act like hormones, while others act like anti-hormones. In either case, they disrupt normal endocrine physiology.

An endocrine disruptor of much concern is bisphenol A [BPA] because it is widely dispersed in the environment due to the presence of BPA in polycarbonate plastics, which are used in containers for food and water, including baby bottles, as well as the linings of metal cans used for food and beverages [8], [9], [10]. Leaching of the BPA monomer from these sources into food, milk and the environment exposes humans [11], [12], [13] and wildlife [2], [14] to BPA.

A consequence of the widespread use of BPA is that over 90% of the general population is exposed to BPA [9], [13], [15]. BPA levels range from 0.3 nM to 40 nM in maternal plasma and fetal human serum [8], [10], [11]. Moreover, due to the lipophilic nature of BPA, it can accumulate in fat [16].

**Figure 1:**
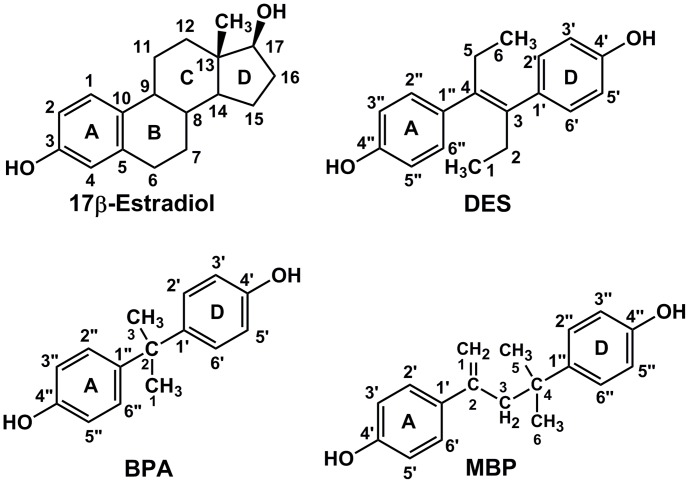
Structures of MBP, BPA, E2 and DES. MBP, BPA and DES have a phenolic ring that can mimic the A ring on E2 in binding to ERα and ERβ. The spacing between the first and second phenolic hydroxyls on MBP and DES is similar to that between C3 hydroxyl and the 17β-hydroxyl on E2. In contrast, the distance between the two phenolic hydroxyls in BPA is shorter than that in E2.

BPA has some structural similarity to estradiol and diethylstilbestrol [Figure 1], and, indeed, BPA binds to human estrogen receptor α [ERα] and ERα and is a transcriptional activator of these ERs [17], [18], [19], [20]. However, BPA’s binding affinity and transcriptional activity for these ERs is over 1000-fold lower than that of E2 [17], [18], [19], [20], which makes it unlikely that nM concentrations of BPA would disrupt estrogen physiology. Nevertheless, *in vivo* studies indicate that BPA is active at 1 nM to 10 nM [8], [10], [15], [21], which raises the possibility that BPA is metabolized to a more active endocrine disruptor. One such candidate metabolite is 4-methyl-2,4-bis(4-hydroxyphenyl)pent-1-ene [MBP] [Figure 1], which has about 1000-fold higher estrogenic activity than BPA [22], [23]. To begin to understand the structural basis for the high estrogenic activity of MBP and its higher affinity compared to BPA for human ERα and ERβ, we constructed 3D models of MBP and BPA in human ERα and ERβ. We find that MBP retains key contacts with human ERα and ERβ that are important in activation of these receptors by estradiol. We also find that one phenolic ring of BPA can mimic binding of the A ring of E2 to ERα and ERβ, which would account for the binding of BPA to these ERs. However, the second phenolic ring on BPA lacks some key contacts that are found between E2 and both ERs, which may explain the lower estrogenic activity of BPA. In addition to elucidating the interaction of MBP and BPA with both human ERs, this analysis may be useful in designing novel chemicals for regulating the actions of human ERα and ERβ.

## Methods

Human ERα [24] was downloaded from the Protein Data Bank [PDB] as a template for docking of MBP and BPA. ChemDraw 3D was used to create PDB files for MBP and BPA, which were docked to human ERα [PDB:1G50] with AutoDock 4 [25], [26] and AutoDock Vina [27]. The grid was centered over the estrogen binding site in human ERα. AutoDock 4 was run using the Lamarckian Genetic Algorithm for 250 trials of 5 million energy evaluations. AutoDock Vina was run with a setting of 20 for exhaustiveness and poses for the 100 lowest energies were collected.

The crystal structure of ERβ complexed with E2 [PDB:3OLS] [28] was selected for docking MBP and BPA. As was found in other ERβ structures in the PDB, 3OLS lacks coordinates for five amino acids corresponding to residues 416–420. To model the missing amino acids, we used the Homology option in Insight II and the 1G50 structure for human ERα as a template. A PDB file of the complete ERβ with E2 was refined with Discover 3 with the CVFF force field and a distant dependent dielectric constant of 2 for 50 iterations. We docked MBP and BPA into this PDB file of human ERβ with AutoDock 4 and AutoDockVina [27] with the settings used previously for human ERα.

**Figure 2:**
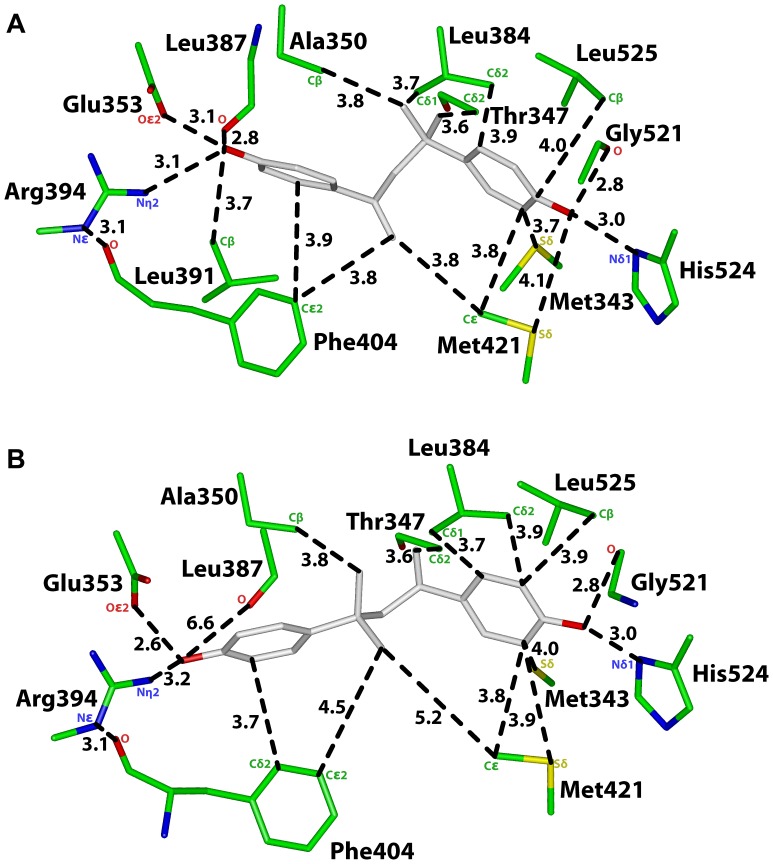
Analysis of two 3D models of MBP in human ERα. A. 3D model of MBP in orientation 1 in human ERα. The first phenolic ring on MBP contacts Glu-353, Arg-394 and Phe-404 on ERα and the second phenolic ring contacts Gly-521, His-524 and Leu-525. Favorable van der Waals contacts have a distance of 4.25 Å or less between MBP and amino acids on ERα. **B.** 3D model of MBP in orientation 2 in human ERα. The first phenolic ring on MBP contacts Glu-353, Arg-394 and Phe-404 on ERα, and the second phenolic ring contacts Gly-521, His-524 and Leu-525. However, in contrast to Orientation 1, the backbone oxygen on Leu-387 does not contact the phenolic hydroxyl on MBP. Phe-404 and Met-421 do not have van der Waals contacts with the linker between the two phenolic rings on MBP.

**Table 1:** Distances between MBP and ERα.

Figure 2A	ERα	MBP	Distance
Orientation 1	Oε2, Glu-353	O4’	3.1 Å
	Nη2, Arg-394	O4’	3.1 Å
	O, Leu-387	O4’	2.8 Å
	Cβ, Leu-391	O4’	3.7 Å
	Cε2, Phe-404	C2’	3.9 Å
	Cε2, Phe-404	C1	3.8 Å
	Nδ1, His-524	O4”	3.0 Å
	O, Gly-521	O4”	2.8 Å
	Cβ, Leu-525	C4”	4.0 Å
	Sδ, Met-343	C3”	3.7 Å
	Sδ, Met-421	O4”	4.1 Å
	Cε, Met-421	C3”	3.8 Å
	Cδ2, Leu384	C6	3.9 Å
	Cδ1, Leu384	C6	3.7 Å
	Cδ2, Thr-347	C5	3.6 Å
	Cβ, Ala-350	C6	3.8 Å
**Figure 2B**	**ERα**	**MBP**	**Distance**
Orientation 2	Oε2, Glu-353	O4”	2.6 Å
	Nη2, Arg-394	O4”	3.2 Å
	O, Leu-387	O4”	6.6 Å
	Cβ, Leu-391	O4”	3.7 Å
	Cδ2, Phe-404	C3”	3.9 Å
	Cε2, Phe-404	C6	4.5 Å
	Nδ1, His-524	O4’	3.0 Å
	O, Gly-521	O4’	2.8 Å
	Cβ, Leu-525	C3’	3.9 Å
	Sδ, Met-343	C5’	4.0 Å
	Sδ, Met-421	C5’	3.9 Å
	Cε, Met-421	C5’	3.9 Å
	Cε, Met-421	C6	5.2 Å
	Cδ1, Leu384	C2’	3.7 Å
	Cδ2, Leu384	C3’	3.9 Å
	Cδ2, Thr-347	C1	3.6 Å
	Cβ, Ala-350	C5	3.8 Å

**Figure 3:**
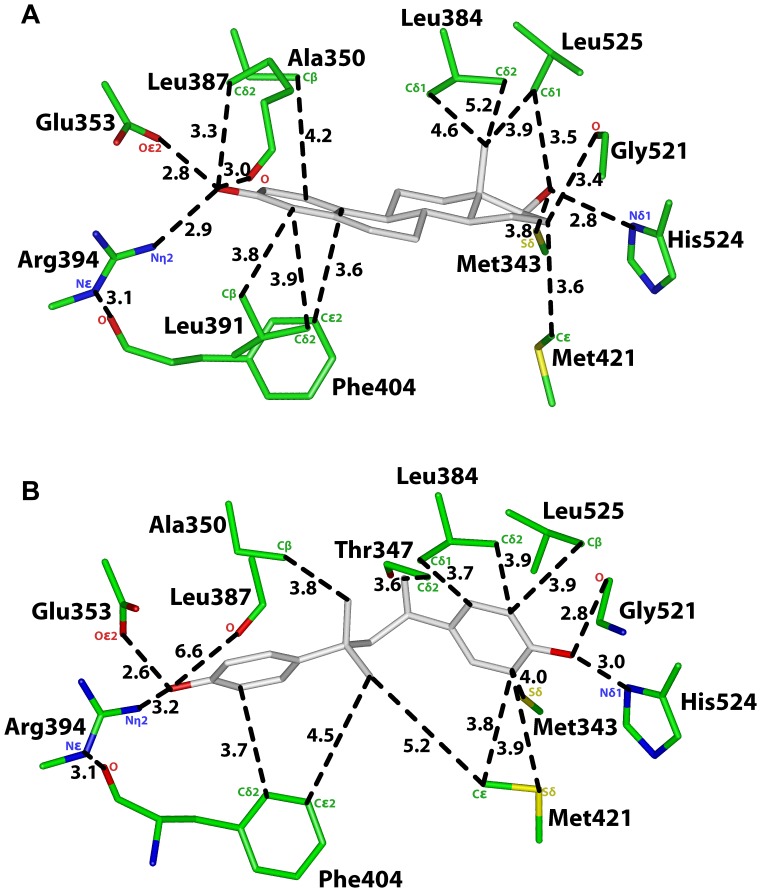
Interaction of E2 with amino acids in human ERα and ERβ. A. Interaction of E2 with human ERα [24], [33], [34], [36], [37], [38], [39]. The phenolic hydroxyl of E2 contacts Glu-353, Arg-394 and Leu-387. The 17β-hydroxyl contacts His524 and Leu-525. The D ring contacts Met343, Met421, Gly-521 and Ile-424. Favorable van der Waals contacts have a distance of 4.25 Å or less between E2 and amino acids on ERα. **B.** Interaction of E2 with human ERβ [28]. The phenolic hydroxyl of E2 contacts Glu-305, Arg-346 and Leu-339. The 17β-hydroxyl contacts Gly-472, His473 and Leu-476. The D ring contacts Met-336 and Ile-373. Favorable van der Waals contacts have a distance of 4.25 Å or less between E2 and amino acids on ERβ.

**Table 2:** Distances between E2 and ERα and ERβ.

Figure 3A	ERα	E2	Distance
Crystal Structure	Oε2, Glu-353	O3	2.8 Å
PDB: 1G50	Nη2, Arg-394	O3	2.9 Å
	O, Leu-387	O3	3.0 Å
	Cδ2, Leu-387	O3	3.0 Å
	Cε2, Phe-404	C10	3.6 Å
	Nδ1, His-524	O17	2.8 Å
	O, Gly-521	O17	
	O, Gly-521	C16	3.4 Å
	Cδ1 Leu-525	O17	3.5 Å
	Cδ1 Leu-525	C18	3.9 Å
	Sδ, Met-343	O17	3.8 Å
	Cε, Met-421	O16	3.6 Å
	Sδ, Met-421	O17	
	Cδ2, Leu384	C18	5.2 Å
	Cδ1, Leu384	C18	4.6 Å
	Cβ, Ala-350	C1	4.2 Å
	Cβ, Leu-391	C4	3.8 Å
	Cδ2, Leu-391	C4’	3.9 Å
**Figure 3B**	**ERβ**	**E2**	**Distance**
Crystal Structure	Oε2, Glu-305	O3	2.6 Å
PDB: 3OLS	Nη2, Arg-346	O3	3.0 Å
	O, Leu-339	O3	3.4 Å
	Cβ, Leu-339	C2	4.0 Å
	Cδ1, Leu-343	C4	4.0 Å
	Cδ2, Leu-343	C4	3.9 Å
	Cε2, Phe-356	C5	3.7 Å
	Cβ, Ala-302	C1	3.9 Å
	O, Gly-472	O17	3.9 Å
	Nδ1, His-475	O17	3.0 Å
	Cβ, Leu-476	O17	3.4 Å
	Cδ2 Leu-476	C18	3.9 Å
	Cε, Met-295	O17	3.5 Å
	Sδ, Met-336	C18	3.7 Å
	Cε2, Met-336	C18	3.4 Å
	Cδ1, Ile-373	C16	3.8 Å
	Cε, Met-421	O17	3.5 Å

The lowest energy complexes of MBP and BPA in ERα and ERβ, as calculated by AutoDock 4 and AutoDock Vina, were refined with the Discover 3 software in Insight II. For this energy minimization step, Discover 3 was used with the CVFF force field and a distant dependent dielectric constant of 2 for 10,000 iterations. During this refinement step, both the amino acids on the ERs and MBP and BPA rearrange their positions so as to lower the Gibbs free energy of the complex.

### Docking Energy Analysis

We used X-Score [29], [30] and DSX [DrugScore eXtended] [31] to estimate the relative binding energy of MBP and BPA in the various configurations in ERα and ERβ. X-Score uses an empirical scoring function to estimate the affinity of a ligand for a protein. DSX uses a knowledge-based scoring function based on the DrugScore formalism [32] to estimate the affinity of a ligand for a protein. In comparing the score of two ligands for a protein, the ligand with the larger negative score has the higher affinity.

**Figure 4:**
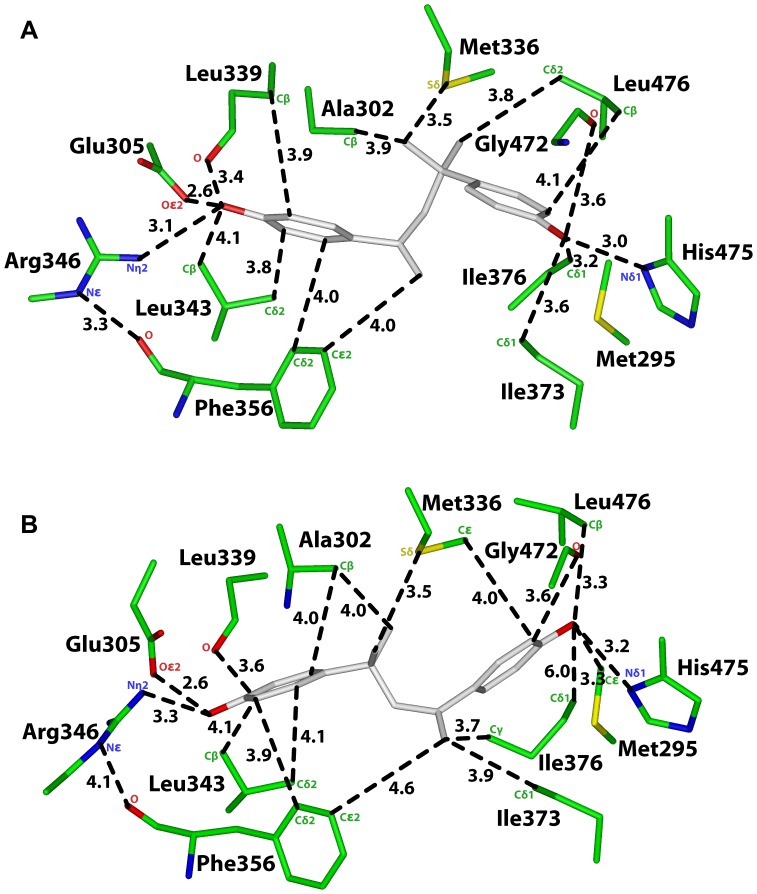
Analysis of two 3D models of MBP in human ERβ. A. 3D model of MBP in orientation 1 in human ERβ. The first phenolic ring on MBP contacts Glu-305, Arg-346, Leu-339, Leu-343 and Phe-356. The second phenolic ring contacts Gly-472, His-475 and Leu-476, which are important in the interaction of the D ring of E2 with ERβ. **B.** 3D model of MBP in orientation 2 in human ERβ. The first phenolic ring on MBP contacts the backbone oxygen on Leu-339, Cβ on Ala-302 and Leu-343. These contacts are absent between MBP in Orientation 2 in ERα [Figure 2B].

**Table 3:** Distances between MBP and ERβ.

Figure 4A	ERβ	MBP	Distance
Orientation 1	Oε2, Glu-305	O4’	2.6 Å
	Nη2, Arg-346	O4’	3.1 Å
	O, Leu-339	O4’	3.4 Å
	Cβ, Leu-343	O4’	3.9 Å
	Cδ2, Phe-356	C2’	4.0 Å
	Cε2, Phe-356	C1	4.0 Å
	Nδ1, His-475	O4”	3.0 Å
	O, Gly-472	O4”	3.6 Å
	Cβ, Leu-476	C3”	4.1 Å
	Cε, Met-295	O4”	5.7 Å
	Cβ, Ala-302	C6	3.9 Å
	Sδ, Met-336	C6	3.5 Å
	Cδ1, Ile-373	O4”	3.6 Å
	Cδ1, Ile-376	O4”	3.2 Å
**Figure 4B**	**ERβ**	**MBP**	**Distance**
Orientation 2	Oε2, Glu-305	O4”	2.6 Å
	Nη2, Arg-346	O4”	3.3 Å
	O, Leu-339	C4”	3.6 Å
	Cβ, Leu-343	C5”	4.1 Å
	Cδ2, Leu-343	C6”	4.1 Å
	Cδ2, Phe-356	C5”	3.9 Å
	Cε2, Phe-356	C1	4.6 Å
	Nδ1, His-475	O4’	3.2 Å
	O, Gly-472	C5’	3.6 Å
	Cβ, Leu-476	O4’	3.3 Å
	Cε, Met-295	O4’	3.3 Å
	Cβ, Ala-302	C5	4.0 Å
	Cβ, Ala-302	C2”	4.0 Å
	Sδ, Met-336	C6	3.5 Å
	Cε, Met-336	C5’	4.0 Å
	Cδ1, Ile-373	C1	3.9 Å
	Cγ, Ile-376	C1	3.7 Å
	Cδ1, Ile-376	O4’	6.0 Å

## Results

### Docking of MBP and BPA to Human ERα and ERβ

Docking of MBP into human ERα and ERβ using AutoDock 4 [25], [26] and AutoDock Vina [27] gave two symmetric poses, which is not surprising because MBP has a phenolic ring at each end [Figure 1]. BPA also had two poses for one of the rings in ERα and ERβ. We analyzed both poses for MBP and BPA in human ERα and ERβ. In our analysis of the 3D models of MBP and BPA in both ERs, we use the term “first phenolic ring” to describe the ring that has contacts with ERα and ERβ that are similar to the A ring of E2.

**Figure 5:**
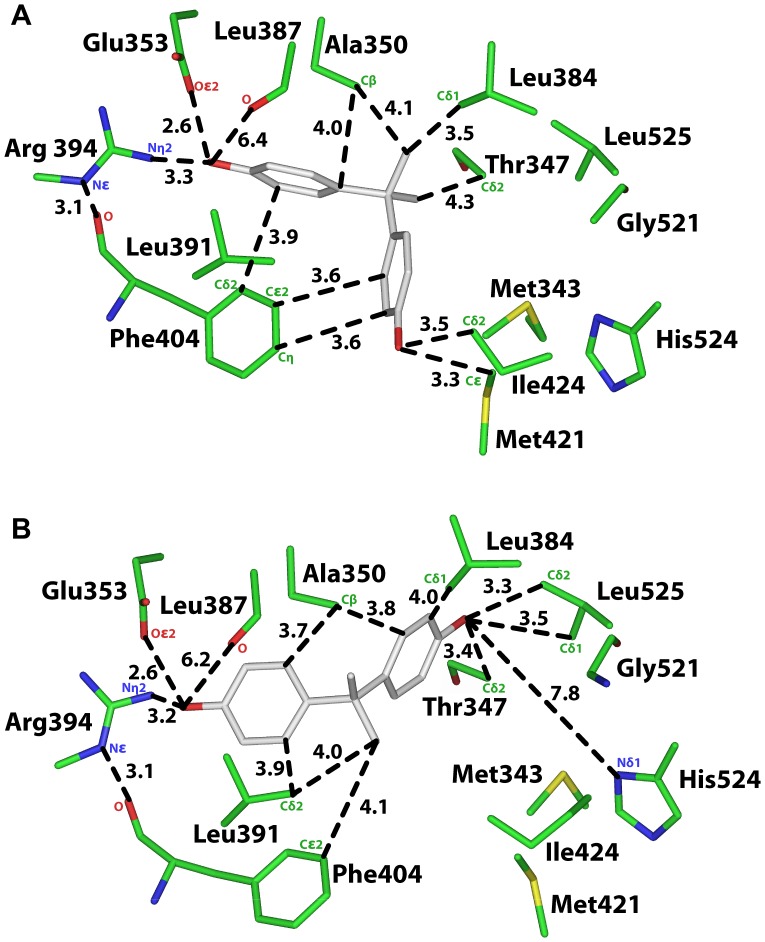
Analysis of two 3D models of BPA in human ERα. A. 3D model of BPA in orientation 1 in human ERα. The first phenolic ring on BPA contacts Glu-353, Arg-394 and Phe-404 on ERα, but does not contact either Leu-387 or Leu-391. Moreover, the second phenolic ring does not contact either Gly-521, His-524 or Leu-525. Instead, the second phenolic ring contacts Phe-404, Met-421 and Ile-424. **B.** 3D model of BPA in orientation 2 in human ERα. The first phenolic ring on BPA contacts Glu-353 and Arg-394 on ERα, but does not contact Leu-387 or Phe-404. The second phenolic ring does not contact either Gly-521 or His-524. Instead, the second phenolic ring has novel contacts with Thr-347 and Leu-384.

**Table 4:** Distances between BPA and ERα.

Figure 5A	ERα	BPA	Distance
Orientation 1	Oε2, Glu-353	O4’	2.6 Å
	Nη2, Arg-394	O4’	3.3 Å
	O, Leu-387	O4’	6.4 Å
	Cδ2, Phe-404	C3’	3.9 Å
	Cε2, Phe-404	C6”	3.6 Å
	Cη, Phe-404	C5”	3.6 Å
	Nδ1, His-524	O4”	6.8 Å
	Cδ1, Leu-384	C1	3.5 Å
	Cβ, Ala-350	C1	4.1 Å
	Cβ, Ala-350	C1’	4.0 Å
	Cε, Met-421	O4”	3.3 Å
	Cδ2, Ile-424	O4”	3.5 Å
	Cδ2, Thr-347	C3	4.3 Å
**Figure 5B**	**ERα**	**BPA**	**Distance**
Orientation 2	Oε2, Glu-353	O4’	2.6 Å
	Nη2, Arg-394	O4’	3.2 Å
	O, Leu-387	O4’	6.2 Å
	Cδ2 Leu-391	C6’	3.9 Å
	Cδ2 Leu-391	C3	4.0 Å
	Cε2, Phe-404	C3	4.1 Å
	Nδ1, His-524	O4”	7.8 Å
	O, Gly-521	O4”	7.4 Å
	Cβ, Leu-525	O4”	3.3 Å
	Cδ1, Leu-525	O4”	3.5 Å
	Sδ, Met-343	C5”	5.7 Å
	Sδ, Met-421	C5”	7.7 Å
	Cε, Met-421	C5”	7.6 Å
	Cε, Met-421	C6”	7.1 Å
	Cδ1, Leu384	C3”	4.0 Å
	Cδ2, Thr-347	O4”	3.4 Å
	Cβ, Ala-350	C2”	3.8 Å
	Cβ, Ala-350	C2’	3.7 Å

Analysis of the crystal structures of ERα complexed with E2 [33], [34] and other estrogens [35] revealed that Glu-353 and Arg394 have important stabilizing contacts with the C3 hydroxyl on the A ring and His-524 with the 17β-hydroxyl on the D ring. Glu-305, Arg-346 and His-475 on ERβ have similar stabilizing contacts with estrogens. As reported below, the presence or absence of these contacts in the 3D models of ERα and ERβ with BPA and MBP is important analyzing the interaction between these chemicals and the ERs.

### Analysis of MBP in Orientation 1 in Human ERα

In Figure 2A, we show the 3D model of MBP in human ERα in Orientation 1, in which C1 on MBP is closest to first phenolic ring. The distances between MBP and ERα are shown in Figure 2A and Table 1. For comparison, in Figure 3A and Table 2 we show the distances between E2 and human ERα [33], [35], [36], [37], [38].

**Figure 6:**
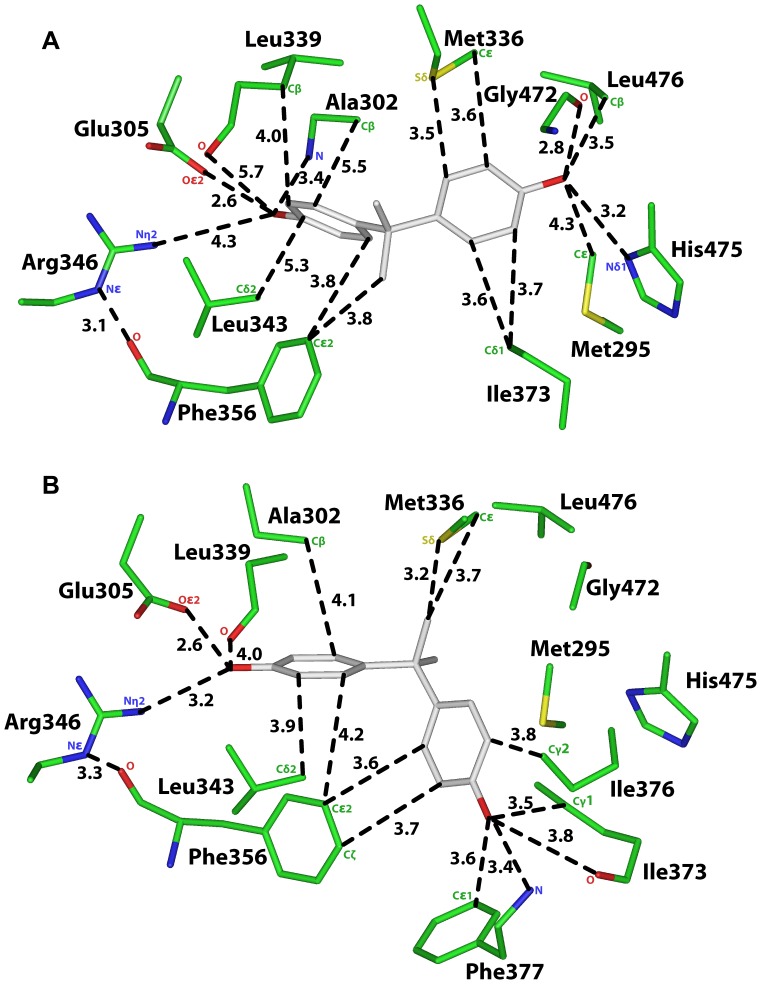
Analysis of two 3D models of BPA in human ERβ. A. 3D model of BPA in orientation 1 in human ERβ. The first phenolic ring on BPA contacts Glu-305, Arg-346, and Phe-356, but does not contact either the backbone oxygen on Leu-339 or Cδ2 on Leu-343. The second phenolic ring contacts Gly-472, His-475 and Leu-476. **B.** 3D model of BPA in orientation 2 in human ERβ. The first phenolic ring on BPA contacts Glu-305, Arg-346, Phe-356, the backbone oxygen on Leu-339 and Cδ2 on Leu-343. The second phenolic ring does not contact either Gly-472, His-475 or Leu-476.

**Table 5:** Distances between BPA and ERβ.

Figure 6A	ERβ	BPA	Distance
Orientation 1	Oε2, Glu-305	O4’	2.6 Å
	Nη2, Arg-346	O4’	4.3 Å
	O, Leu-339	O4’	5.7 Å
	Cβ, Leu-339	O4’	4.0 Å
	Cδ2, Leu-343	C4’	5.3 Å
	Cδ2, Phe-356	C3’	4.0 Å
	Cε2, Phe-356	C2’	3.8 Å
	Cε2, Phe-356	C3	3.8 Å
	Nδ1, His-475	O4”	3.2 Å
	O, Gly-472	O4”	2.8 Å
	Cβ, Leu-476	O4”	3.5 Å
	Cε, Met-295	O4”	4.3 Å
	Cβ, Ala-302	C6’	5.5 Å
	N, Ala-302	O4’	3.4 Å
	Sδ, Met-336	C2”	3.5 Å
	Cε, Met-336	C3”	3.6 Å
	Cδ1, Ile-373	C5”	3.7 Å
	Cδ1, Ile-376	C6”	3.6 Å
**Figure 6B**	**ERβ**	**BPA**	**Distance**
Orientation 2	Oε2, Glu-305	O4’	2.6 Å
	Nη2, Arg-346	O4’	3.2 Å
	O, Leu-339	C4’	4.0 Å
	Cδ2, Leu-343	C5’	3.9 Å
	Cε2, Phe-356	C6’	4.2 Å
	Cε2, Phe-356	C6”	3.6 Å
	Cζ2, Phe-356	C5”	3.7 Å
	Nδ1, His-475	O4”	7.1 Å
	O, Gly-472	O4”	8.4 Å
	Cβ, Leu-476	O4”	10.2 Å
	Cε, Met-295	O4”	8.5 Å
	Cβ, Ala-302	C2’	4.1 Å
	Sδ, Met-336	C3	3.2 Å
	Cε, Met-336	C3	3.7 Å
	Cδ1, Ile-373	C1	3.9 Å
	Cγ1, Ile-376	O4”	3.5 Å
	O, Ile-376	O4”	3.8 Å
	Cε1, Phe-377	O4”	3.6 Å
	N, Phe-377	O4”	3.4 Å

The first phenolic ring on MBP has contacts that are similar to that of the A ring on E2 with human ERα [33], [34], [39]. The phenolic hydroxyl on MBP is 3.1 Å from Oε2 on Glu-353, 3.1 Å from Nη2 on Arg-394 and 2.8 Å from the backbone oxygen of Leu-387. MBP is 3.9 Å from Cε2 on Phe-404 [Figure 2A, Table 1]. These contacts are similar to that for E2 with human ERα, except that Cδ2 on Leu-387 does not contact the phenolic hydroxyl on MBP, in contrast to the contact between Leu-387 and E2 in human ERα [Figure 3A, Table 2].

**Table 6:** Docking analysis of MBP and BPA in ERα and ERβ.

Receptor	Ligand	Score	Figure
ERα	E2	7.4	Figure 3A
ERα	MBP Orientation 1	7.2	Figure 2A
ERα	MBP Orientation 2	7.2	Figure 2B
ERα	BPA Orientation 1	6.5	Figure 4A
ERα	BPA Orientation 2	6.5	Figure 4B
ERβ	E2	7.5	Figure 3A
ERβ	MBP Orientation 1	7.1	Figure 5A
ERβ	MBP Orientation 2	7.2	Figure 5B
ERβ	BPA Orientation 1	6.7	Figure 6A
ERβ	BPA Orientation 2	6.7	Figure 6B

X-Score Analysis of MBP and BPA in ERα and ERβ [29], [30].

**Table 7:** Docking analysis of MBP and BPA in ERα and ERβ.

Receptor	Ligand	Score	Figure
ERα	E2	−116	Figure 3A
ERα	MBP Orientation 1	−103	Figure 2A
ERα	MBP Orientation 2	−110	Figure 2B
ERα	BPA Orientation 1	−84	Figure 4A
ERα	BPA Orientation 2	−91	Figure 4B
ERβ	E2	−123	Figure 3B
ERβ	MBP Orientation 1	−112	Figure 5A
ERβ	MBP Orientation 2	−116	Figure 5B
ERβ	BPA Orientation 1	−90	Figure 6A
ERβ	BPA Orientation 2	−89	Figure 6B

DSX Analysis of MBP and BPA in ERα and ERβ [31], [32].

The second phenolic hydroxyl in MBP is 3 Å, 2.8 Å and 4 Å from Nδ1 on His-524, the backbone oxygen on Gly-521 and Cβ on Leu-525, respectively, on ERα [Figure 2A]. This phenolic hydroxyl also contacts Met-343 and Met-421 on ERα. These five residues stabilize the D ring on E2 in human ERα [Figure 3A].

There are, however, differences in some interactions between ERα and MBP compared to that with E2. While Gly-521 and Met-421 contact the second phenolic hydroxyl on MBP [Figure 2A], Gly-521 and Met-421 contact C16 on E2 in ERα [Figure 3A]. While Leu-384 has two van der Waals contacts with MBP, Leu-384 does not contact E2 in ERα. While Thr-347 has a van der Waals contact with MBP, Thr-347 does not contact E2 in ERα. While Cβ on Leu-391 is 3.7 Å from the first phenolic hydroxyl on MBP, this contact is absent between ERα and E2. While Ala-350 contacts the linker between the two phenolic rings on MBP, Ala-350 contacts C1 on the A ring in E2 in ERα. Phe-404 and Met-421 have van der Waals contacts with C1 on MBP, which has no equivalent in E2 in ERα.

**Figure 7:**
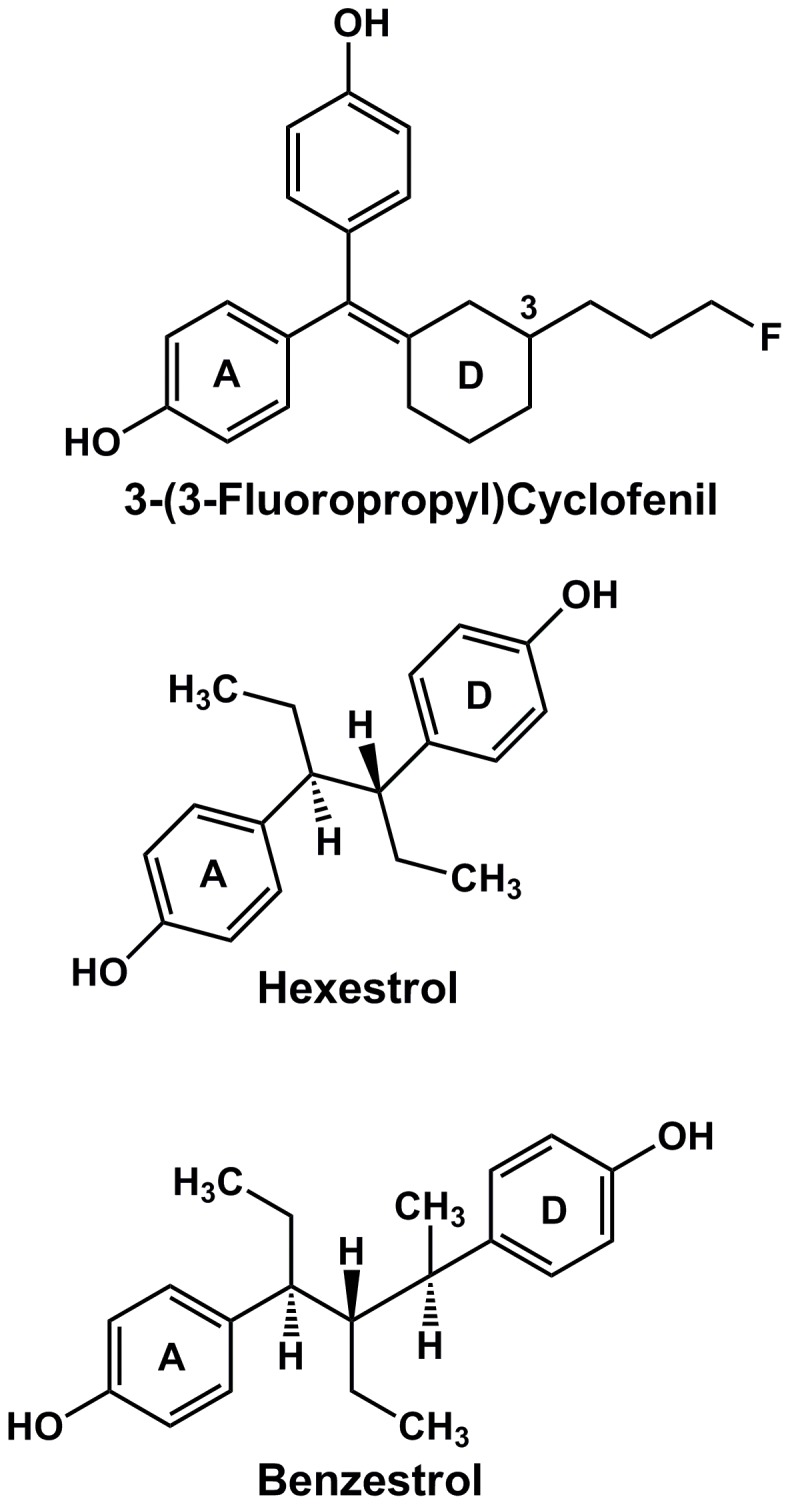
Structures of bisphenols that are potent synthetic estrogens. Bisphenols, linked with one, two or three carbons, can have high affinity for ERs. 3-(3-fluoropropyl)cyclofenil, hexestrol and benzestrol have a higher affinity for ERα that does E2 [18], [39], [40].

### Analysis of MBP in Orientation 2 in Human ERα

As shown in Figure 2B and Table 1, analysis of ERα with MBP in Orientation 2 reveals that MBP has contacts with Glu-353, Arg-394, Phe-404, Met-343, Leu-384, Met-421, Gly-521, His-524 and Leu-525 that are similar to those found in Orientation 1 of MBP in ERα. Due to the reversed orientation of MBP in ERα, C1 on MBP has a van der Waals contact with Thr-347, and the other part of the linker contacts Ala-350.

### Analysis of MBP in Orientation 1 in Human ERβ

Figure 4A shows MBP in Orientation 1 in human ERβ. For comparison, in Figure 3B, we show E2 in human ERβ [28]. Many of the contacts between MBP and human ERβ shown in Figure 4A and Table 3 are similar to that between MBP in Orientation 1 and human ERα [Figure 2A, Table 2] and between E2 and ERβ [Figure 3B]. Like the A ring in E2, the first phenolic ring on MBP has stabilizing contacts with Glu-305, Arg-346, Phe-356, Leu-339 and Leu-343 in ERβ. The second phenolic ring contacts His-475, Gly-472, Leu-476, Ile-373 and Ile-376 [Figure 4A, Table 3].

### Analysis of MBP in Orientation 2 in Human ERβ

Figure 4B shows MBP in Orientation 2 in human ERβ. Many of the contacts between MBP in Orientation 2 and human ERβ [Table 3] are similar to that between MBP in Orientation 1 and human ERβ [Figure 4A, Table 3]] and between E2 and ERβ [Table 2]. The backbone oxygen on Leu-339, Cβ on Ala-302 and the side chains on Leu-343 contact the first phenolic ring on MBP. These contacts are absent between MBP in Orientation 2 in ERα [Figure 2B, Table 1].

### Analysis of BPA in Orientation 1 in Human ERα

Figure 5A shows BPA in Orientation 1 in human ERα. The phenolic ring on BPA, corresponding to the A ring of E2, has stabilizing contacts with Oε2 on Glu-353, Nη2 on Arg-394, Cδ2 on Phe-404 and Cβ on Ala-350 [Figure 5A, Table 4]. However, the backbone oxygen on Leu-387 is 6.4 Å from the phenolic hydroxyl and Leu-391 does not have a van der Waals contact with the phenolic ring.

The second phenolic ring does not contact either Gly-521, His-524 or Leu-525 [Table 4]. Instead, phenolic ring moves so that it contacts Cε2 and Cη on Phe-404, Cε on Met-421 and Cδ2 on Ile-424. Also, Ala-350 and Leu-384 and Thr-347 contact the linker on BPA.

### Analysis of BPA in Orientation 2 in Human ERα

In Figure 5B, we show BPA in Orientation 2 in human ERα. The first phenolic ring on BPA contacts Oε2 on Glu-353, Nη2 on Arg-394, Cδ2 on Phe-404 and Cβ on Ala-350 [Figure 5B, Table 4]. Leu-391 has a van der Waals contact with the phenolic ring. However, the backbone oxygen on Leu-387 does not contact BPA.

The second phenolic ring on BPA does not contact either Gly-521, His-524, Met-421 or Ile-424 [Table 4]. Instead, the phenolic hydroxyl contacts Leu-525 and Thr-347. Leu-384 and Ala-350 also contact the second phenolic ring.

### Analysis of BPA in Orientation 1 in Human ERβ

In Figure 6A, we show BPA in Orientation 1 in human ERβ. The first phenolic ring on BPA contacts Oε2 on Glu-305, Nη2 on Arg-346, Cε2 on Phe-404, Cβ on Leu339 and the backbone nitrogen on Ala-302 [Figure 6A, Table 5]. The backbone oxygen on Leu-339 does not contact the phenolic hydroxyl. Cβ on Ala-302 and Cδ2 on Leu-343 do not contact the phenolic ring.

The second phenolic ring contacts Gly-472, His-475, Leu-476, Ile-373 and Met-336, but the second phenolic ring does not contact Met-295.

### Analysis of BPA in Orientation 2 in Human ERβ

In Figure 6B, we show the minimized structure of BPA in Orientation 2 in human ERβ. The first phenolic ring on BPA contacts Oε2 on Glu-305, Nη2 on Arg-346, Cε2 on Phe-404, Cβ on Ala-350, Cδ2 on Leu-343 and the backbone oxygen on Leu-339 [Figure 6B, Table 5].

The second phenolic ring does not contact either Gly-472, His-475, Leu-476 or Met-295 [Table 5]. Instead, the phenolic hydroxyl contacts Phe-377 and Ile-373. Interestingly, Phe-356 contacts the second phenolic ring and Met-336 contacts the linker on BPA.

### Docking Energy Analysis

We used X-Score [31] and DSX [31] to estimate the affinity of MBP and BPA in their different orientations in ERα and ERβ. Tables 6 and 7 summarize these analyses for the X-Score and DSX. For both algorithms, MBP has an affinity for ERα and ERβ that is closer to that of E2 than is BPA for these receptors. This is consistent with previous assays of the activity of MBP and BPA [22], [23].

## Discussion

The leaching of BPA monomers from polycarbonate containers and from liners of metal containers for food and beverages has contributed to the widespread exposure of humans to BPA [2], [11], [12], [13], [15]. The relatively low affinity of BPA, compared to E2, for human ERα and ERβ [17], [19], [20] would, at first glance, make it unlikely that BPA would be a problem as an estrogenic endocrine disruptor at nM concentrations [9], [10], [11]. However, it is clear that nM concentrations of BPA have estrogenic activity [8], [21]. The discovery that MBP, a metabolite of BPA, has a nM affinity for human ERα and ERβ, suggests that metabolism of BPA to MBP could explain some of effects of BPA on estrogen physiology [22], [23].

There is a structural basis for considering BPA and MBP as potential ligands for ERα and ERβ because BPA and MBP have some structural similarities to known synthetic estrogens [Figure 7]. BPA is a bisphenol linked by one carbon atom [Figure 1] as are cyclofenil-type estrogens [Figure 7], some of which have high affinity for ERα and ERβ [40]. MBP is a bisphenol linked by three carbon atoms [Figure 1] as is benzestrol [Figure 7], which has a high affinity for ERα and ERβ [39]. Hexestrol, which is linked by two carbon atoms, also has a high affinity for ERα and ERβ [18], [39]. Thus, it is reasonable to be concerned about potential endocrine disruption by synthetic bisphenols. However, as discussed below, our 3D model of BPA in ERs indicates that BPA does not have the contacts with an ER as is found between fluorine-substituted cyclofenil derivatives [40]. Studies with a wide variety of synthetic bisphenols [39], [40] indicate the length of the carbon linker between bisphenols and side chain substitutents on the cyclohexane ring on cyclofenils are important in establishing contacts that lead to high affinity binding to the ER. This is consistent with the analysis of our 3D models of BPA and MBP in ERα and ERβ as discussed below.

### MBP Retains Important Contacts found between E2 and ERα and ERβ

Our 3D models of MBP and BPA in human ERα and ERβ [Figures 2, 4–6] identify contacts that can explain MBP’s high affinity and BPA’s low affinity for both estrogen receptors. A key structural difference between BPA and MBP is the longer spacing between the two phenolic rings in MBP [Figure 1]. As a result, both phenolic rings on MBP form stabilizing contacts with ERα and ERβ that are similar to that between the A and D rings of E2 and human ERα and ERβ [28], [33], [34], [35], [38], [39], [41]. These 3D models predict that the second phenolic hydroxyl on MBP has a hydrogen bond with His-524 on ERα and His-475 on ERβ. Our 3D models can be tested by investigating transcriptional activation by MBP of ERα and ERβ in which His-524 and His-475, respectively, have been mutated.

### BPA Lacks some Contacts found between E2 and ERα and ERβ

Like the A ring on E2, one phenolic ring on BPA has stabilizing contacts with Glu-353, Arg-394 and Phe-404 in ERα [Figure 5]. Interestingly, Phe-404 also contacts the second phenolic ring. However, the second phenolic ring on BPA does not contact either Gly-521 or His-524 on ERα, which is significant because contacts between E2 and Gly-521 and His-524 in ERα are important in binding of E2 [28], [33], [35], [39], [41]. Also, Leu-387 does not contact the first phenolic ring of BPA in either Orientation 1 or Orientation 2. The loss of these contacts between BPA and ERα may explain the lower affinity of BPA for ERα.

In the 3D model of BPA in Orientation 1 in ERβ [Figure 6A], one phenolic ring on BPA has stabilizing contacts with Glu-305, Arg-346 and Phe-356 in ERβ. Moreover, the second phenolic ring on BPA contacts Gly-472, His-475 and Leu-476 on ERβ. Thus, some of the key interactions between the ERβ and the A and D rings on E2 [Figure 3B] are conserved for BPA in Orientation 1 in ERβ. However, neither Leu-339 nor Leu-343 contacts the first phenolic ring on BPA. The loss of these contacts would be expected to lower affinity of BPA for ERβ.

Although BPA in Orientation 2 in ERβ [Figure 6B] has contacts with Glu-305, Arg-346, Phe-356, Leu-339 and Leu-343, BPA does not contact either Gly-472, His-475 or Leu-476 on ERβ. Instead, the second phenolic ring has novel contacts with Ile-373, Ile-376, Phe-377 and Phe-356.

### Cellular Context Influences Estrogenic Activity of MBP and BPA

The presence of two phenolic rings in MBP and BPA and the flexibility in the estrogen binding site in ERα and ERβ [7], [33], [35], [42], [43], [44], [45], [46] are important factors in the binding of MBP and BPA to these ERs. The equilibrium dissociation constant of BPA for ERα and ERβ is about 195 nM and 35 nM respectively [18]. Using a different binding assay, IC50s [23] for binding of BPA and MBP to ERα and ERβ were reported. BPA and MBP have IC50s of 1.8 µM and 52 nM, respectively for ERα and IC50s of 0.74 µM and 0.12 µM, respectively, for ERβ.

Also important for transcriptional activation of ERs and other nuclear receptors by steroids and endocrine disruptors is the binding of co-regulators to the ligand-receptor complex [47], [48], [49], [50], [51], [52], [53]. Thus, even a low affinity ligand such as 27-hydroxy-cholesterol can have transcriptional activity for the ER in the presence of the appropriate co-activator [44].

The interaction of complexes of ERα and ERβ with different co-regulators may explain the report by Yoshihara et al. [23] that transcriptional activation of ERα and ERβ by MBP and BPA depended on the cellular context. That is, the estrogenic activity of MBP and BPA is altered in the presence or absence of co-regulators [20], [48], [50], [51], [54], [55]. In the yeast estrogen screening (YES) assay, the EC50 potencies for transcriptional activation of ERα by MBP and BPA were 0.7 µM and 160 µM, respectively. In experiments, which included the Transcriptional Intermediary Factor 2 [TIF2] co-activator in the assay, the EC50s for transcriptional activation of rat ERα with TIF2 by MBP and BPA were 8.3 nM and 14 µM, respectively, and the EC50s for rat ERβ with TIF2, by MBP and BPA were 8.3 nM and 13 µM, respectively. Thus, transcriptional activation of ERs by MBP and BPA in an assay containing TIF2, which mimicked conditions in some mammalian cells, increased by about 10-fold compared to the assay in yeast cells.

Further evidence for the importance of cellular context on transcriptional potency of MBP and BPA comes from experiments using an ERE-luciferase reporter assay in 3T3 cells. Yoshihara et al. [23] found that the EC50s for MBP and BPA for ERα were 0.68 nM and 1 µM, respectively. For ERβ in 3T3 cells, the EC50s for MBP and BPA were 0.46 nM and 89 nM, respectively. Together these experiments by Yoshihara et al. suggest that MBP is a potential disruptor of physiological responses that are mediated by ERα and ERβ.

Although MBP and benzestrol have a three carbon linker between their two phenols, their linkers are different. Despite this difference, nM concentrations of MBP activate transcription of the ER in mammalian cells. This raises the possibility that other environmental chemicals with two phenolic rings connected with novel aliphatic linkers may have a physiologically relevant activity towards ERα and ERβ in cells with co-activators that can activate the chemical-ER complex. We also note that the 3D models of MBP in ERα and ERβ may be useful in the development of new chemicals for use as selective ER agonists or antagonists.
